# Communication in the context of glioblastoma treatment: A qualitative study of what matters most to patients, caregivers and health care professionals

**DOI:** 10.1177/02692163231152525

**Published:** 2023-02-03

**Authors:** Florien W Boele, Sean Butler, Emma Nicklin, Helen Bulbeck, Lucy Pointon, Susan C Short, Louise Murray

**Affiliations:** 1 Leeds Institute of Medical Research, St James’s Hospital, University of Leeds, Leeds, UK; 2 Leeds Institute of Health Sciences, University of Leeds, Leeds, UK; 3 Brainstrust – The Brain Cancer People, Leeds, UK; 4 Leeds Institute of Medical Research, School of Psychology, University of Leeds, Leeds, UK

**Keywords:** Brain tumour, glioblastoma, caregivers, communication, decision-making, palliative care

## Abstract

**Background:**

Patients with glioblastoma have a poor prognosis and treatment is palliative in nature from diagnosis. It is therefore critical that the benefits and burdens of treatments are clearly discussed with patients and caregivers.

**Aim:**

To explore experiences and preferences around glioblastoma treatment communication in patients, family caregivers and healthcare professionals.

**Design:**

Qualitative design. A thematic analysis of semi-structured interviews.

**Setting/participants:**

A total of 15 adult patients with glioblastoma, 13 caregivers and 5 healthcare professionals were recruited from Leeds Teaching Hospitals NHS Trust.

**Results:**

Four themes were identified: (1) *Communication practice and preferences*. Risks and side-effects of anti-tumour treatments were explained clearly, with information layered and repeated. Treatment was often understood to be ‘the only option’. Understanding the impact of side-effects could be enhanced, alongside information about support services. (2) *What matters most*. Patients/caregivers valued being well-supported by a trusted treatment team, feeling involved, having control and quality of life. Healthcare professionals similarly highlighted trust, maintaining independence and emotional support as key. (3) *Decision-making*. With limited treatment options, trust and control are crucial in decision-making. Patients ultimately prefer to follow healthcare professional advice but want to be involved, consider alternatives and voice what matters to them. (4) *Impact of COVID-19*. During the pandemic, greater efforts to maintain good communication were necessary. Negative impacts of COVID-19 were limited, caregivers appeared most disadvantaged by pandemic-related restrictions.

**Conclusions:**

In glioblastoma treatment communication, where prognosis is poor and treatmentwill not result in cure, building trusting relationships, maintaining a sense of control and being well-informed are identified as critical.

**What is already known about the topic?**

- Patients with glioblastoma have a very poor prognosis and limited survival. Different from other types of primary neoplasm, glioblastomas manifest also as a neurological disease. Therefore, palliative care of patients with glioblastoma represents a difficult challenge for healthcare professionals and caregivers since it has to be directed to both general and neurological cancer symptoms.
- Obtaining in-depth knowledge of patient and caregiver experiences of communication around treatment can help to improve clinical services, palliative and supportive care and even impact positively on patients’ and caregivers’ psychological burden.

**What this paper adds?**

- In what is a palliative and poor prognosis scenario, patients require better information about the real life side-effects of anti-tumour treatment, supportive medication and supportive services.
- More patients and caregivers want involvement in decision-making, and greater access to information makes them better able to participate in clinical decisions.
- For effective communication, building trusting relationships, maintaining a sense of control and being well-informed are identified as critical by patients, caregivers and healthcare professionals.

**Implications for practice, theory or policy**

- Clinical teams must take time to provide in-depth information about active treatment but also alternative options including exploring experimental treatment, and best supportive and palliative care.
- In light of COVID-19 and with the rise of remote consultations, we should be aware of the associated limitations and barriers to effective communication, such as patients finding remote consultations less reassuring and the reduced capacity to provide information resources.
- Tailored information resources should be modified and/or developed to help patients understand about potential treatment side-effects and supportive services.

## Introduction

Glioblastoma is the most common primary malignant brain tumour i, accounting for 49.1% of malignant cases in adults.^1^ Patients are not only confronted with cancer, but experience neurological symptom burden, with, for example, cognitive deficits, seizures and communication deficits impacting on everyday life.^2^ Prognosis is poor, with most patients surviving less than 1 year.^3^ Treatment is palliative in nature from the point of diagnosis, aimed at delaying disease progression and managing symptoms/preserving quality of life. Even patients who receive optimal therapy at initial diagnosis (debulking surgery, chemo-radiotherapy and adjuvant chemotherapy), glioblastoma almost always recurs after 6–9 months.^4^ The best treatment for glioblastoma at recurrence is unknown, and interventions may include surgery, nitrosourea-based chemotherapy regimens, re-irradiation or best supportive and palliative care, depending on the individual case.

Given the poor prognosis, it is important that the benefits and risks of treatment options are clearly explained to patients and caregivers. The respective value of quantity versus quality of life varies for each individual. However, after a cancer diagnosis, it can be difficult for patients and caregivers to process complex information fully.^5,6^ Recall of information provided in clinical consultations is known to be poor, particularly in highly distressing situations.^7^ Patients’ and caregivers’ awareness of prognosis can vary.^8,9^ Understanding treatment risks and benefits can be further complicated by neurocognitive deficits, common in patients with glioblastoma.^2^ Patients with glioblastoma and their caregivers have a need for individualised information on diagnosis and progosis.^10^ Indeed, how patients and caregivers understand communication about prognostic information and how oncologists recall discussions, does not always align.^11^ Yet, better patient-centred information provision is associated with better health related quality of life and less anxiety and depression.^12^

Since the COVID-19 pandemic, oncology services have been impacted, with social distancing guidelines and personal protective equipement (PPE – refering to protective clothing e.g. gloves, face masks, goggles designed to protect the wearer from the spread of infection) potentially affecting communication between patients, caregivers and healthcare professionals. This study aimed to gain insight into patient, caregiver and healthcare professionals experience of communication around treatment, including the impact of the COVID-19 pandemic. The results can be used to improve communication practices so that patients with glioblastoma and caregivers can have more informed and patient-centred discussions regarding treatment and palliative care options.

## Methods

### Study design

This was a qualitative study using semi-structured interviews and thematic analysis, underpinned by a reflexive approach.^13,14^ We took a interpretivist-constructivist paradigm^13,15^ to explore how patients with glioblastoma, their caregivers and healthcare professionals experienced and made sense of communication around palliative care (during COVID-19) whilst recognising the importance of researcher influence in such interpretations. The study was reported following the Consolidated Criteria for Reporting Qualitative Research guidelines.^16^

### Setting and population

Participants were recruited from Leeds Teaching Hospitals NHS Trust during July 2021–January 2022. Adult (⩾18) patients were eligible if they had a histologically confirmed glioblastoma. Patients were excluded if their treating physician believed they had severe cognitive dysfunction impeding their ability to complete study procedures. Adult family caregivers were eligible if they were a close family member/friend of an eligible patient. Adult healthcare professionals were eligible if involved in the care of patients with glioblastoma. All eligible participants had to speak and understand English and be willing to provide informed consent.

### Sample

We used convenience and purposive sampling techniques to obtain a sample of patients and caregivers representing different disease stages (e.g. newly diagnosed, undergoing treatment, in follow-up), and healthcare professionals representing different clinical backgrounds.

### Recruitment

Eligible patients and caregivers were identified by the treatment team, then approached by a researcher during a follow-up visit or by telephone. Eligible healthcare professionals were approached via email. All participants were given detailed verbal and written study information and provided written or recorded verbal consent.

### Interviews

Interviews were conducted by telephone or video-call, dependent on participant preference. Healthcare professionals took part individually. Patients and caregivers took part individually, unless they preferred to be interviewed together. A semi-structured interview guide (Supplemental Information 1), explored experiences around glioblastoma treatment. Interviews were performed by an experienced qualitative research assistant (LP, PhD candidate) and postdoctoral research fellow (EN, PhD), supervised by FWB (PhD), none of whom are involved in patient care. Data collection stopped when researchers felt theoretical saturation was achieved, meaning we believed we had reached a sufficient depth of understanding to build a theory and address the research questions.^17^ Interviews were audio-recorded and detailed field notes written after each interview.

### Ethical considerations

The study was approved by the East Midlands Nottingham Research Ethics Committee in February 2021 (21/EM/0006). Interviews covered sensitive topics with potentially vulnerable participants. However, the research team are experienced in conducting research with people living with cancer. A plan for any participants who experienced distress during the interviews included stopping or pausing the interview, and providing details of further support (e.g. their clinical team or GP).

### Data analysis and reporting

Interviews were transcribed smooth verbatim, and analysed using reflexive thematic analysis.^13–15^ Two coders (SB-male and FWB-female) read the transcript several times to familiarise themselves with the contents, before highlighting sections. Coding was inductive to fully understand participant communication experiences but also deductive to find data to address the research objectives. Each coder independently identified codes from participant responses and confirmed agreement. The initial codes were subsequently categorised into potential sub-themes and themes. The coders met frequently to discuss their findings, refine the key issues and themes and resolve possible differences until consensus was reached. Themes were also discussed with the broader research team (EN, LM and SS) to enable in-depth interpretation before being finalised.

## Results

### Participants

In total, 19 patients, 20 caregivers and 5 healthcare professionals were approached with study information. A 16 patients (84%), 16 caregivers (80%) and 5 healthcare professionals (100%) agreed to participate. Prior to interview, one patient and three caregivers withdrew, due to lack of time or disease progression. In total, 15 patients, 13 caregivers (*N* = 12 individual and *N* = 8 dyadic interviews) and 5 healthcare professionals participated (*N* = 5 individual interviews). Interviews took on average 49 min (range 26–99). See Table 1 for participant characteristics.

**Table 1.** Participant characteristics.

	Patients (*N* = 15) (%)	Caregivers (*N* = 13) (%)	Healthcare professionals (*N* = 5) (%)
Age			
31–40	0 (0)	2 (15.4)	2 (40.0)
41–50	4 (26.7)	0 (0)	3 (60.0)
51–60	2 (13.3)	0 (0)	0 (0)
61–70	6 (40.0)	8 (61.5)	0 (0)
71–80	3 (20.0)	2 (15.4)	0 (0)
Unknown	0 (0)	1 (7.7)	0 (0)
Sex			
Male	5 (33.3)	7 (53.8)	3 (60.0)
Female	10 (66.7)	6 (46.2)	2 (40.0)
Ethnicity			
White British or Irish	14 (93.3)	8 (61.5)	4 (80.0)
British Asian	0 (0)	0 (0)	1 (20.0)
White (mixed or other)	1 (6.7)	2 (15.4)	0 (0)
Unknown	0 (0)	3 (23.1)	0 (0)
Relationship with patient			
Spouse or partner	N/a	11 (84.6)	N/a
Friend	N/a	1 (7.7)	N/a
Child	N/a	1 (7.7)	N/a
Time since diagnosis (years)			
<1	8 (53.3)	N/a	N/a
1–2	4 (26.7)	N/a	N/a
>2	3 (20.0)	N/a	N/a
Treatments received			
Surgery (biopsy)	3 (20.0)	N/a	N/a
Surgery (debulking)	12 (80.0)	N/a	N/a
Radio- and chemotherapy (concurrent/adjuvant, Full course*	10 (66.7)	N/a	N/a
Radio- and chemotherapy (concurrent/adjuvant, Short course**)	2 (13.3)	N/a	N/a
Chemotherapy only (first line)	1 (6.7)	N/a	N/a
Chemotherapy (second/third line)	7 (46.7)	N/a	N/a
Disease status at time of interview			
Between surgery and start of radio-/chemotherapy	2 (13.3)	N/a	N/a
Under treatment	9 (60.0)	N/a	N/a
In follow-up	3 (20.0)	N/a	N/a
Formal referral to Palliative Care Team in place at time of interview			
Yes	5 (33.3)	N/a	N/a
No	10 (66.6)		
Healthcare professional role			
Disease progression	1 (6.7)	N/a	N/a
Clinical oncologist			2 (40.0)
Clinical nurse specialist			2 (40.0)
Neurosurgeon			1 (20.0)

Four over-arching themes each associated with sub-themes, were constructed from the interview data are discussed in detail below and displayed in Table 2.

* Full course, Stupp regimen, ** Short course, Perry regimen – generally reserved for patients ⩾70 years old.^18^

**Table 2.** Themes and sub-themes constructed from interview data.

Themes	Sub-themes
1. Communication practices and preferences	- Barriers to communication include: rush to treatment, brain tumour-specific symptoms, shock and patients/caregivers not knowing whom to question.
	- Side-effects of anti-tumour treatment are clearly relayed but communication could be improved regarding real-life impact, supportive medication and support services.
2. What matters most?	- Building a trusting relationship with care team.
	- Weighing up treatment options and alternatives.
	- Preserving health related quality of life.
	- Understanding the next steps to aid planning ahead.
3. Decision making	- Patients and caregivers want to follow healthcare professional advice and they want to:
	○ Be involved in decision-making, and voice what matters to them
	○ Know whether treatment is the best or the only option.
	○ Maintain some sense of control.
4. Impact of COVID-19	- Remote consultations can hamper communication, be less interactive and reassuring, as non-verbal information is lost.
	- Social distancing and PPE further hinders effective communication and support.

### Communication practice and preferences

Across the treatment pathway patients and caregivers reported receiving information through a variety of methods (mainly verbal, supported by written). Patients and caregivers felt surgical options were explained clearly, with scans used to support verbal explanations. Barriers to communication at this early stage include shock and brain tumour specific symptoms such as confusion and memory problems, the urgency for treatment, but also not knowing who to ask:

It was the speed of it. So we went to see the neurosurgeon on Wednesday, and I was under the knife on the Friday. [Male patient about to start chemo- and radiotherapy.]

*I felt like a lot of the questions that we had weren’t necessarily relevant to the surgeon at that point? But we wanted to talk to somebody.* [Female caregiver of patient about to start chemo-and radiotherapy]

Healthcare professionals acknowledge the rush and limited surgical options, but thought patients generally appreciated swift actions. While risks of surgical treatment were clearly explained, some patients felt risks were overemphasised and not balanced against potential benefits. Most patients reported feeling like they had little choice but to agree with surgery. Some expressed for better written information about surgical options, which could help when feeling overwhelmed during consultations.

Histomolecular diagnosis was communicated by surgeons supported by nurses. Generally, patients and caregivers were satisfied with this approach and valued the honesty and matter-of-factness of consultants in combination with the support from nurse specialists.

Following surgery, healthcare professionals started ‘layering’ information to prepare patients for further treatment. The pace of information provision, offering information at multiple time points and revisiting were tailored to aid understanding:

*The nurse specialists will come along and say on the Monday afternoon that this particular family - I don’t think they particularly understood what was said, or they were just too traumatised by the news that they really didn’t take very much on board last week. So we often have that kind of heads up from the nurse specialists, if it’s going to be a slightly non-standard consultation where you’re going to have to go back a few steps.* [Male clinical oncologist.]

Regardless of treatment modality, managing expectations around risks and benefits of treatment was considered crucial by all stakeholders. Reconciling realistic treatment outcomes in a poor prognosis and palliative situation with the desire to maintain hope was difficult. In communication of treatment options, chemo- and/or radiotherapy were often interpreted as ‘the only option’, with the alternative being no treatment. Exploring experimental treatment was frequently raised by all stakeholders but best supportive or palliative care were not mentioned during interviews. This was also true for patients with progressive disease:

*They wouldn’t do it [surgery], because of the risks, so the only option open to me was to have treatment via tablets really.* [Male patient on second line treatment]

Prior to starting chemo- and/or radiotherapy, patients and caregivers generally report that potential side-effects were communicated clearly. Yet, during and after treatment patients and caregivers explained that they could not have anticipated the real-life impact of side-effects, highlighting an opportunity for improved communication:

*I don’t think at that time you’re necessarily in a position to really understand when somebody says fatigue, what that really means.* [Female caregiver of patient in follow-up.]

Patients and caregivers praised the treatment team for good responsiveness, valuing continuity in team members. On occasions when healthcare professionals were difficult to reach, they could become distressed:

*My only problem with the oncology nurses is that they’re not instantly available if you have a problem. Because you actually make a telephone call, which they then put on an answering machine, and then you’re not actually talking to a person. They ring you back, but not necessarily that day. But your problem is your chemotherapy hasn’t arrived, then you can’t wait another day.* [Female patient on third line treatment.]

Patients and caregivers were less satisfied with communication around side-effects from supportive medication, including corticosteroids, which could be serious and distressing:

*I’ve ended up in the oncology wing for. . .uncontrolled diabetes, which was steroid induced. They didn’t warn me about that, and we weren’t monitoring for it.* [Male patient on second line treatment.]

Many patients and caregivers mentioned examples of information and support they had received, but some explained that communication about support services could be more proactively provided:

*I had to request that [support service], and did on the basis that my friend saying you need support and a community. . . I wouldn’t have known that that existed at all if she hadn’t told me, and nobody offered it.* [Female caregiver of patient on second line treatment.]

### What matters most?

Patients and caregivers said that having reliable and clear information, and good support from the treatment team was crucial. They also wanted to involve the caregiver throughout. Healthcare professionals similarly emphasised that open and honest conversations about options, however limited, are important. Healthcare professionals felt that discussing whether treatment has worked, what the alternatives might be in terms of experimental or second line, whilst providing emotional support was crucial:

*It’s about being honest and saying this is our next standard treatment, yes there are trials available possibly, but we have to think about whether that’s suitable, whether you’re eligible, I think it’s about being realistic. . .keeping the patients and the families aware of what is and isn’t possible.* [Male clinical oncologist.]

Patients and caregivers also mentioned the importance of understanding treatment procedures, and what the next steps are, so they could plan life around it:

*The most important thing to me is to know the kind of timescales I’ve got for each individual thing that’s going to happen and what sort of state I’m going to be in. . .So I can plan what I need to do in work and that sort of thing.* [Female patient about to start radio- and chemotherapy.]

Not only was planning everyday life important, but planning for the end of life was also mentioned by a patient to alleviate anxieties around death and dying. Healthcare professionals acknowledged how important support and reassurance is, particularly in supporting caregivers:

*A lot of our role is supporting families and just acknowledging and listening, and acknowledging that this is rubbish. We can’t change the situation or make it any better. . .but we can be there to listen and support. [*Female nurse specialist.]

All stakeholders frequently mentioned the importance of building a trusting relationship with the treatment team:

*Trust is incredibly important isn’t it, I think that the thing that really struck me when we first went was how it felt like very personable care, people took an interest in her as individuals, and us*. [Female caregiver of patient in follow-up.]

*We have to develop a relationship built with honesty and trust, so that people feel that they are getting the best care they can in this awful situation that they can ask us things and they believe the answers.* [Male clinical oncologist.]

Within the context of treatment outcome, all stakeholders valued extending life when health related quality of life could be preserved. They valued having a good understanding of prognosis whilst maintaining a sense of hope. Caregivers regularly mentioned that spending time together, with the patient being awake and aware, was more important than length of life:

*If they’d have come to us and said – you can have 18 months but the last 6 months will be pretty crap or 12 months of party party party – that’s what we would have done.* [Bereaved male caregiver.]

Caregivers expressed some different needs from patients. Caregivers stressed the importance of continuity of care to help them navigate patient care and support. In terms of their own wellbeing, they valued not feeling isolated, and could struggle to balance the different roles they now had – as family member, and as caregiver.

### Decision-making

A trusting relationship with the treatment team is important in supporting treatment decision-making. Ultimately, patients and caregivers generally followed healthcare professionals’ advice. However, they did want to be involved in decision-making, and be able to voice what matters to them and their families:

*I trusted the medical staff to know what they were doing, they do this day in day out. . .. So I trusted them and I was led, but I also did feel in control to say no. . ..I could have said no but I didn’t* [Female patient in follow-up.]

Patients and caregivers wanted to know what their options are, whether a treatment is the best or only option. They expressed feeling desperate to grasp any chance for better outcomes, and are often keen to discuss alternative or experimental treatments:

*[Patient name] had picked up some research by a clinic that was being run in London and probably the information was right but the consultant just said “it’s not been proven its not been clinically tested, we’ve seen no results from anything they’ve been doing, we can’t tell you if there will be any benefits or not, it will be expensive so if I was you I just wouldn’t bother”. [Patient name] was, not clutching at straws but looking to see what was out there.* [Bereaved male caregiver.]

Healthcare professionals expressed wanting to support patient wishes, and empowering them to make informed decisions. Healthcare professionals see their role as managing expectations in view of uncertain outcomes, and monitoring ability to cope and capacity to give consent. Healthcare professionals see shared decision-making as involving all parties:

*For glioblastoma is there is no right or wrong answer, there’s no right or wrong treatment for anybody. So there’s no idea at the beginning of how well these patients will do, it’s what the patient wants to do, and with support of their families.* [Female nurse specialist.]

Having a feeling of being in control remained important for patients and caregivers. They want to have a say in deciding to stop or pause treatment:

*I would have had another round of treatment this week. . .but we’ve agreed to pause it for a couple of weeks because I’m going on holiday. I don’t really want to feel more poorly than I already do when I’m on holiday*. [Male patient on second line treatment.]

Healthcare professionals acknowledge that there are limited opportunities for patients to feel in control. They manage this by offering opportunities to seek a second opinion, and by providing patients and caregivers with time.

### Impact of COVID-19

During the COVID-19 pandemic, routine consultations were performed over the phone. Patients and caregivers felt remote consultations were less reassuring, explaining they could struggle to contribute and got less out of these, missing non-verbal cues. Remote consultations reduced opportunities to build a trusting relationship with the treatment team:

*When we were getting contact on the phone. . .I wasn’t convinced and I wasn’t reassured that. . .it was stable. . .I mean if we’d been face-to-face they could have shown me the actual scan, all up on screen.* [Male caregiver of patient on second line treatment.]

For routine check-ups, many patients were content with remote consultations and appreciated the time saved. Healthcare professionals could identify positive aspects of remote consultations (convenient, faster) but echoed patient and caregiver concerns about the potential to miss non-verbal information, and not having caregiver input:

*Sometimes I don’t know if patients feel confident enough to tell you everything over the phone, sometimes it’s very different when you have the patient physically in front of you. They maybe open up more, or don’t feel so rushed, and obviously you don’t have the visual cues.* [Male oncologist.]

Only bringing patients to clinic for bad news raised concerns in healthcare professionals, especially after prolonged remote consultations, causing patient anxiety as changing to face-to-face might signal deterioration.

*If we carry on doing telephone consultations at some point this tumour grows back, how do you then communicate that that’s gone from telephone to face-to-face, without that patient then thinking this is because it’s bad news?* [Female nurse specialist.]

For face-to-face appointments, patients often stated how having a caregiver present was crucial for their understanding and mental wellbeing. While this was usually allowed, guidelines for bringing caregivers were unclear and inconsistent, causing confusion. Social distancing and PPE were seen as understandable and necessary, but could also impact communication. Masks particularly removed opportunities to read lips or facial expressions. Healthcare professionals echoed these barriers, and also found PPE to constrain patient expressions and the level of support they could offer, for example, being unable to hold someone’s hand.

## Discussion

### Main findings

Interviews with patients with glioblastoma, caregivers and healthcare professionals yielded important insights into communication around treatment. Discussions about treatment and palliative care are particularly challenging in the setting of glioblastoma due to high symptom burden and progressive cognitive deficits. What matters most to patients and caregivers is having a trusting relationship with their clinical team, being well-informed of treatment options to be able to contribute in decision-making, maintaining some sense of control and being able to preserve quality of life. Barriers to effective communication included the rush to treatment, brain tumour-specific symptoms (e.g. memory loss and confusion), shock and patients/caregivers not knowing whom to question. All stakeholders felt the COVID-19 pandemic restrictions did hamper effective communication and support, having implications on the future of remote consultations. While our study represents a unique population, findings may be useful in other populations suffering from similar symptom burden or poor prognosis.

### What this study adds?

Our findings highlight experiences and preferences in communication about treatment and decision-making, what matters most to patients and caregivers and how communication was affected by pandemic-related restrictions. Throughout these themes, three overarching narratives are identified: building trusting relationships; maintaining a sense of control; and being well-informed.

Trust in healthcare is crucial, forming the foundation of interpersonal relationships.^19^ It is associated with better patient satisfaction, continuity of care and improved outcomes.^20,21^ It drives effective communication and decision-making.^22,23^ Nonverbal communication plays a critical role of building a competence-based trust alliance between patients and caregivers, and their healthcare professionals – yet nonverbal communication was invariably mentioned by our participants as affected by COVID-19 measures. A study in breast cancer patients looked at the role of different aspects of nonverbal communication (eye contact, body posture and smiling) and found that consistent eye contact paryiculalry was associated with better trust.^24^ This is a hopeful message as PPE still allowed eye contact for those seen in clinic, and thus potentially limited the impact on forming trusting relationships. However, remote consultations in our brain tumour clinics are commonly done via telephone rather than video-calls, removing all aspects of nonverbal communication with a potential negative impact on trust.

Participants emphasised the importance of having some form of control. The diagnosis comes unexpectedly, prognosis is poor, there is a need to move quickly at first presentation and effective anti-tumour treatment options are limited. In advanced cancer people can make adjustments to the focus of control.^25^ Our study highlights opportunities for patients and caregivers to maintain a greater sense of control: supporting them to plan life around treatments, explore experimental treatment options and reserving the option to stop treatment. Our interviews highlighted that for all stakeholders, it is important to weigh quality and quantity of life. However, in considering treatment options this was presented as, or *interpreted* as, anti-tumour treatment versus no treatment. This does not do justice to the evidence-base on the benefits of supportive and palliative care, including longer survival and improved quality of life.^26,27^ Our finding is similar to previous studies that highlighted that despite high symptom burden, only a minority of high-grade glioma patients interact with palliative care.^28^ Palliative care services are often postponed until the last weeks or days of life.^29^ However, research supports that curative and palliative intervention should overlap in time.^26,30^ Screening tools aimed at the success of treatment but also query palliative care-related need, may help identify patients who would benefit from early palliative care.^31^ Family caregivers can also experience benefits of early palliative care, reducing caregivers depressive symptoms and burden.^32^

Using a different methodological approach could have yielded different results. An analysis of 60 audio-recorded consultations about palliative chemotherapy, starting with preference-related talk about starting treatment, was found to hinder coverage of patient appraisals and values. When oncologists show empathy, check patients’ views and use probe questioning, preference-related conversations can enhance shared-decision making.^33^ In the context of glioblastoma and palliative care, this can be complicated by progressive cognitive deficits and limited capacity to consent. Therefore, it seems important to involve patients with glioblastoma early in disease trajectory in treatment decision-making. Advance care planning could enable patients and their caregivers to plan future (palliative) care, including end of life care.^34^ Evidence supports that advance care planning can significantly reduce hospital readmission rates and admission to intensive care.^35^ However, it is still unclear the optimal timing for advance care planning.^36^

The need for patients with glioblastoma and caregivers to be well-informed and supported is recognised.^7,37,38^ Our study confirms this also applies when communicating about treatment options and in decision-making. This is again interlinked with building trust, through continuity of care and staff availability. Although not highly novel, it is important to note that participants in our study would appreciate more proactive communication about support services. Patients’ understanding about potential side-effects and supportive services could be improved by providing tailored information resources.

### Strengths and limitations of the study

Strengths of the current investigation include the in-depth nature of our qualitative interviews, performed in a relatively large sample comprising all stakeholder groups with experience across the glioblastoma trajectory.

Limitations are related to our recruitment methods: as a single-centre study, the results may not be generalisable of other centres’ communication practices; participants were recruited from clinics by invitation of their treating doctor, hence there may be some bias in who participated. Finally, study procedures were adapted to the pandemic-related guidance and were completed remotely, it is possible that face-to-face interviews may have elicited further discussions.

## Conclusions

The present study demonstrates the importance of building trusting relationships between patients, caregivers and healthcare professionals; the value of maintaining a sense of control; and support in communication about treatment options in the palliative and poor prognosis scenario of glioblastoma. Whilst a single-centre study, many of the findings can be applicable to other centres within the UK and internationally, in other conditions where prognosis is poor and treatment palliative, and can be used to enhance communication practice and materials, improving patient-centred care.

## Supplemental Material

sj-pdf-1-pmj-10.1177_02692163231152525 – Supplemental material for Communication in the context of glioblastoma treatment: A qualitative study of what matters most to patients, caregivers and health care professionals Click here for additional data file. Supplemental material, sj-pdf-1-pmj-10.1177_02692163231152525 for Communication in the context of glioblastoma treatment: A qualitative study of what matters most to patients, caregivers and health care professionals by Florien W Boele, Sean Butler, Emma Nicklin, Helen Bulbeck, Lucy Pointon, Susan C Short and Louise Murray in Palliative Medicine
