# Supplementary material for: Communication in the context of glioblastoma treatment: A qualitative study of what matters most to patients, caregivers and health care professionals
Source: Palliat Med. 2023 Feb 3;37(6):834–43. doi: 10.1177/02692163231152525 (PMC10227096; doi:10.1177/02692163231152525)
Supplement: sj-pdf-1-pmj-10.1177_02692163231152525 – Supplemental material for Communication in the context of glioblastoma treatment: A qualitative study of what matters most to patients, caregivers and health care professionals [file sj-pdf-1-pmj-10.1177_02692163231152525.pdf]

**Supporting Information 1**

**Table 1**. Summary interview topic guide, with questions tailored to each stakeholder group (patients, caregivers, healthcare professionals).

| Topics | Key questions |
|---|---|
| 1. **Communication about treatment** | What were your experiences of communication around treatment options? (patients, caregivers) |
| | *How do you typically communicate with patients and caregivers about treatment options? (Healthcare professionals)* |
| | Which treatment benefits and risks matter most to you? (patients, caregivers) |
| | *How do you discuss treatment benefits and risks? (Healthcare professionals)* |
| | What has been your experience with treatment decision-making? (patients, caregiver) |
| | *How are treatment decisions made? (Healthcare professionals)* |
| 2. **Experience of treatment** | What was your experience of treatment received? (patients, caregivers) |
| | *How do patients typically experience treatment? (Healthcare professionals)* |
| | What do you feel was most important to you at that time? (patients, caregivers) |
| | *What do you think is most important to patients and caregivers? (Healthcare professionals)* |
| 3. **Impact of Covid-19** | Has Covid-19 affected communication with your treatment team? If so, how? (patients, caregivers) |
| | *Has Covid-19 affected communication with patients and caregivers? If so, how? (Healthcare professionals)* |
